# Hydrogen ejection from hydrocarbons: Characterization and relevance in soot formation and interstellar chemistry

**DOI:** 10.1073/pnas.2202744121

**Published:** 2024-12-09

**Authors:** Josie Hendrix, Diptarka Hait, Hope A. Michelsen, Martin Head-Gordon

**Affiliations:** ^a^Department of Chemistry, University of California, Berkeley, CA 94720; ^b^Chemical Sciences Division, Lawrence Berkeley National Laboratory, Berkeley, CA 94720; ^c^Department of Chemistry and The PULSE Institute, Stanford University, Stanford, CA 94305; ^d^Department of Mechanical Engineering and Environmental Engineering Program, University of Colorado, Boulder, CO 80309

**Keywords:** polycyclic aromatic hydrocarbons, quantum chemistry, soot formation, interstellar dust formation, resonance-stabilized radicals

## Abstract

Polycyclic aromatic hydrocarbons (PAHs) play a major role in the chemistry of combustion, pyrolysis, and the interstellar medium. Production (or activation) of radical PAHs and propagation of their resulting reactions require efficient dehydrogenation, but the preferred method of hydrogen loss is not well understood. Unimolecular hydrogen ejection (i.e., direct C─H bond fission) and bimolecular radical abstraction are two main candidate pathways. We performed a computational study to characterize the role of H ejection, particularly as a driver for radical-centric hydrocarbon-growth mechanisms and particle formation. Electronic structure calculations establish that C─H bond strengths span a broad range of energies, which can be weaker than 30 kcal/mol in some C_9_ and C_13_ PAH radicals. At *T* > 1200 K, calculated thermal rates for hydrogen ejection from weak C─H bonds at zigzag sites on PAH radicals are significantly larger than typical H-abstraction rates. These results are highly relevant in the context of chain reactions of radical species and soot inception under fuel-rich combustion conditions. Furthermore, calculated microcanonical rates that include the additional internal energy released by bond formation (e.g., ring closure to yield C_9_H_9_) yield significantly higher rates than those associated with full thermalization. These microcanonical considerations are relevant to the astrochemical processes associated with hydrocarbon growth and processing in the low-density interstellar environment.

## Significance

Polycyclic aromatic hydrocarbons (PAHs) play central roles in soot formation and interstellar chemistry. To date, specific pathways to formation of such PAHs are incompletely understood, and there are gaps in our understanding of how PAH molecules grow toward larger carbonaceous particles. How do reactive species form and coalesce during soot and interstellar dust formation? Quantum chemical calculations of bond energies and rate constants reported here establish that unimolecular hydrogen-ejection reactions play a role in the chemical-reaction networks underlying such processes. Rates of H ejection can significantly exceed the rates of H abstraction under many conditions because of strikingly low C─H bond energies in some reactive PAH species believed to be important in soot inception and interstellar dust formation.

Soot particles, composed primarily of carbon and hydrogen, are produced during the incomplete combustion or pyrolysis of hydrocarbons and are released into the environment from sources such as internal combustion engines, coal-powered industries, wildfires, after-harvest burning, and cookstoves common in developing countries (1, 2). Soot particles have detrimental effects on the environment, contributing to worldwide air pollution and global warming (1, 2). They also present a significant hazard to human health, leading to cardiopulmonary and neurological illnesses and deaths (3–6). On the other hand, synthesized soot particles (carbon black) have a variety of useful commercial applications, particularly as a filler to modify material properties (7, 8).

Soot formation begins with the production of molecular precursors, including polycyclic aromatic hydrocarbons (PAHs). These molecular precursors form solid nanoscale (or sometimes larger) particles via processes that are still poorly understood (9–12). For large enough species, growth can occur via physical condensation of large PAHs held together through van der Waals-type forces, but species observed during soot inception are generally too small to explain soot formation by such a mechanism. Alternatively, chemical linkage through covalent-bond formation may explain the inception step associated with formation of the smallest particles (13); however, such mechanisms are typically too slow to reproduce soot formation rates, largely because of high barriers associated with hydrocarbon activation and limitations on abstractor abundances under some conditions (11, 12). Following inception, the structure and stoichiometry of these particles evolve as they undergo surface growth and lose hydrogen to become more carbonized. Incipient particles grow and undergo partial carbonization, eventually forming loosely bound agglomerates (13), which then evolve into tightly bound mature soot particles (referred to as aggregates). The mechanisms for particle growth are also currently unknown (11, 12). The process of particle inception alone is a complex balance of kinetic and thermodynamic factors that existing chemical models cannot fully replicate (11, 12).

**Fig. 1.**
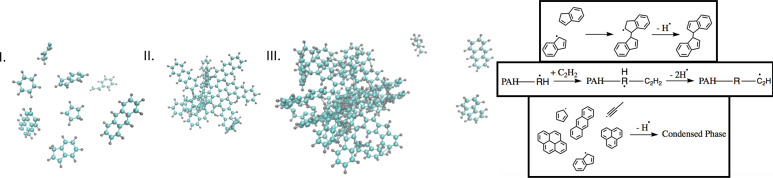
Schematic illustration of the accepted steps initiating soot formation: I) growth of gas-phase precursors, II) particle inception, and III) particle growth. Hot-hydrogen ejection (*Right* inset) may speed reactions in radical-based mechanisms contributing to soot inception and growth (12). This process may also assist dimer stabilization, growth at radical centers, and soot-particle inception.

Fig. 1 depicts these generally accepted steps to begin soot formation. There is an extensive body of experimental and theoretical work on soot precursor formation, inception, growth, and chemical evolution (11, 14) and numerous kinetic models for soot formation (15–18). Despite decades of investigation, however, the detailed mechanisms underlying these processes, particularly for inception, are not yet fully understood (11, 12). The most detailed models include some form of nonspecific nucleation-type mechanism for soot inception (15), but most soot models do not include an explicit soot-inception mechanism, relying instead on nonphysical proxy mechanisms, such as dimerization of pyrene or similar PAHs (16–18). There are conflicting hypotheses for the events leading to (and following) particle inception (Step II in Fig. 1). Most proposed mechanisms involve physical nucleation pathways via stable closed-shell PAHs, e.g., (19–22). Several recent mechanisms have focused on reactive dimerization of large PAHs, but the steps that would lead to inception beyond dimerization are unclear (23–25). An alternative mechanism, the “clustering of hydrocarbons by radical-chain reactions” (CHRCR) (12) mechanism, relies on chemical-bond formation between precursors and is based on the hypothesis that radical-chain reactions involving persistent resonance-stabilized radicals (RSRs) lead to clustering of species while continually regenerating the radical pool. In the CHRCR mechanism, open-shell PAHs serve as clustering centers for particle inception, which is in line with recent evidence that small- to medium-sized PAHs likely contribute to particle inception (12, 26, 27) (in contrast to physical-nucleation mechanisms that require participation of larger PAHs, i.e., ovalene and larger). Although a growing collection of theoretical and experimental evidence supports the CHRCR mechanism (12, 28–34), it has yet to be fully validated.

In CHRCR, growth paths to larger hydrocarbons and hydrocarbon clustering require effective dehydrogenation to continuously regenerate RSRs that propagate the chain reactions leading to inception. What is the preferred method of dehydrogenation for these RSR-fuel adducts? The two principal candidates are bimolecular abstraction by a radical and unimolecular C─H bond fission using internal energy to eject a hydrogen atom.

Molecular-growth models, such as HACA (Hydrogen Abstraction, C_2_H_2_ Addition), widely regarded as an essential mechanism in combustion chemistry, rely on bimolecular abstraction of H atoms from precursor molecules (such as PAHs) by small radicals (12, 35, 36). The rates of such processes are bounded by the collision frequency between the reactant and the abstracting radical. Molecular growth in models involving covalent clustering requires repeated H abstractions, which necessitates persistently high local concentrations of abstractors. Furthermore, the reverse process of radical (often hydrogen) addition to a newly dehydrogenated species occurs at a similar rate as abstraction (36). Experimental results have even suggested that concentrations of small radicals are inversely related to soot-volume fractions under soot-formation conditions (37–39), implying that radicals such as H may directly affect or be affected by soot growth. In hot, dense regions of the flame, where radicals are readily available, abstraction is likely to be the major player; however, in regions where radicals are depleted (e.g., the postflame region, during pyrolysis, or for hydrocarbon growth in extremely sparse environments, such as the interstellar medium), other mechanisms may be needed that do not rely so heavily on two-body collisions.

The alternative dehydrogenation mechanism to abstraction is ejection (unimolecular C─H bond fission) of H atoms from molecules with high internal energy (e.g., hot, or vibrationally excited, or even electronically excited). Such processes dissipate excess energy generated by covalent bond formation. Hydrogen loss from hydrocarbon intermediates during combustion is certainly not a novel process and has been acknowledged in many other combustion mechanisms (e.g., refs. 40–42; however, this essential process rarely receives attention as a key driver of molecular-weight growth and clustering, a role it may perform in mechanisms like CHRCR, as attested by Johansson et al. (12). Ejection of H atoms from hot hydrocarbon molecules is known to lead to significant concentrations of atomic hydrogen in flames (37, 39, 43, 44). Hydrogen elimination through bimolecular reactions and unimolecular hydrocarbon decomposition is a critical component of the pyrolytic cracking of hydrocarbon fuels and is one of the first steps in ignition (39, 45–49). Accurate representation of hydrogen-atom sources is important in combustion models, particularly for predicting soot-precursor concentrations (35) and even for soot surface growth (50); however, reaction inventories included in chemical kinetic combustion models are far from complete. Inclusion of vetted and validated chemical reactions is drastically reduced in models that predict abundances of larger hydrocarbons (C_7_ and above) and soot because of the computational cost and the sheer multitude of viable hydrocarbons that may be present in combustion and pyrolysis environments (51–53). It is thus important to identify and include reactions that impact precursor and particle formation and growth, and characterize dehydrogenation processes for combustion, pyrolysis, and interstellar reactions. Several useful databases have already been compiled for quantities such as bond dissociation energy using large-scale techniques, such as machine learning (e.g., ref. 54). Because the generation of hydrogen atoms and chemical activation of stable species through abstraction (55, 56) are inexorably related, kinetic models for fuel pyrolysis or combustion and soot formation must be able to accurately predict H concentrations under a wide range of conditions.

Efficient dehydrogenation is also imperative for chemical growth reactions in the interstellar medium (ISM). In fact, processes leading to PAHs in combustion environments are often the blueprint for similar processes in the ISM (57). Carbonaceous dust in the outflows of asymptotic giant branch (AGB) stars may form through clustering of PAHs, analogous to soot-particle inception (57, 58). In cold molecular clouds (59, 60) and planetary atmospheres, such as that of Titan (61, 62), PAHs have been detected despite limited sources of energy, low temperatures, and low pressures. Radical-radical and radical-closed-shell reactions involving RSRs are often barrierless or kinetically accessible (58, 63–65) and are likely to contribute to the formation of larger PAHs and H_2_ in the ISM (66). This hypothesis once again begs the question of how PAHs dehydrogenate to form reactive, open-shell species. Studies have shown hydrogen loss is a decomposition pathway following UV excitation in interstellar PAHs (57, 67, 68). In the absence of sufficient abstractors to perform dehydrogenation in the ISM, other processes must be at play to promote radical formation and should be further studied to understand the reaction pathways that lead to interstellar PAHs and their subsequent chemical evolution.

In this work, we revisit H ejection in the context of formation and conversion of hydrocarbons that serve as clustering centers for, or participate in, soot-particle inception or reactions of interstellar RSRs. We report H-ejection rate constants for selected open- and closed-shell hydrocarbon species and analyze how this process compares to H abstraction over a range of temperatures. Specifically, we focus on H ejection in the context of CHRCR pathways involving RSRs associated with (but not limited to) this mechanism and identify and tabulate trends in C─H bond strength and H-ejection rate constants based on hydrocarbon structure, which may be useful for kinetic models. To complete the picture, we examine the role of H ejection in a prototypical CHRCR pathway, modeling the formation of the RSR indenyl, and discuss the competition of various decomposition pathways. Finally, we explore microcanonical and canonical H-ejection rate regimes that may be expected for various sizes of hydrocarbons.

## Results and Discussion

### Variation in CH Bond Strengths.

The stability and lack of reactivity of the C─H bond is the raison d’être for the entire field of C─H activation (69). While C─H bond strength is viewed as a characteristic of the associated bonding environment, values are typically quoted in the range between 85 and 115 kcal/mol for closed-shell molecules (70). The strongest known C─H bond is 133 kcal/mol for acetylene (71, 72). By contrast, radicals sometimes have far lower C─H bond energies, as exemplified by the ethyl radical, at 36 kcal/mol (73, 74). Such bond energies are usually available, but rarely tabulated in a methodical manner for species along a particular reaction pathway. What are the implications for formation and processing of RSR species?

**Fig. 2.**
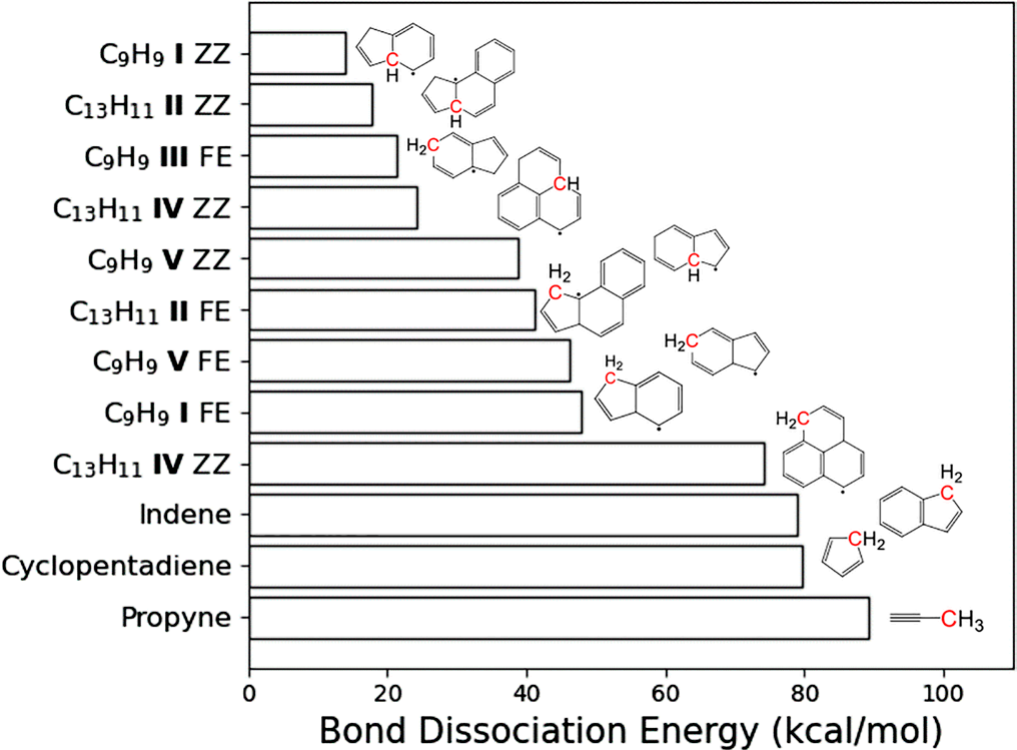
C─H bond-dissociation energies (BDEs) for selected open- and closed-shell parent hydrocarbon species. BDEs are reported for 0 K, i.e., representing the change in electronic and zero-point vibrational energies between the parent and the C─H bond-dissociation products. Isomers are indicated by a Roman numeral. Ejection is examined from free edge (FE) and zigzag (ZZ) sites, indicated in red. Breaking C─H bonds to eject H from closed-shell species is energetically costly, but BDEs are markedly smaller for open-shell species ejecting H to form closed-shell products, diminishing to between 25 and 50% of a saturated C─H bond energy for species **I** to **V**. FE site ejection from **IV** leads to a triplet (and not singlet) hydrocarbon.

Fig. 2 displays the energetic cost (at 0 K) of homolytic C─H bond cleavage for selected conjugated hydrocarbon radicals (C_9_H_9_ and C_13_H_11_ isomers) and closed-shell species (propyne, cyclopentadiene, and indene), showing a tremendous range of bond energies. Notably, a few of these conjugated radicals have C─H bond strengths well below 30 kcal/mol. Several of these species (such as indene and cyclopentadiene) are prominent contributors to combustion reactions (14, 75, 76) and particle formation during combustion and pyrolysis (12, 33, 34). Others are documented or postulated intermediates or precursors leading to species commonly generated during pyrolysis and may give rise to open-shell species important for inception and growth pathways driven by reactions of RSRs. C_13_H_11_ isomers **IV** and **II** are precursors to their respective C_13_H_10_ counterparts 1H-phenalene (77) and cyclopentanaphthalene (78), which have been detected in high yields during pyrolysis experiments (79). Phenalenyl (C_13_H_9_), an RSR indicated to be important for soot inception (12, 33), follows from decomposition of 1H-phenalene (77, 80). Likewise, C_9_H_9_ isomers, such as **I**, **III**, and **V**, form as intermediates in the production of indene (C_9_H_8_) (12, 81, 82), which, in turn, decomposes to form indenyl (C_9_H_7_), another RSR hypothesized to be important in soot inception (12, 34) and interstellar chemistry (12, 66).

The relative stability of R─H and R + H determines C─H bond strength via R─H → R^.^+ H^.^ to create a radical product R^.^ or ^.^R─H → R + H^.^ to yield a closed-shell product R. Well-known factors, such as the degree of substitution at the product radical site, modulate the characteristic C─H bond strengths. The formation of a C─C bond upon fission of the weakest C─H bond in ^.^C_2_H_5_ largely determines its very low bond strength. Aromaticity in the closed-shell product is the key driving force behind the weakest C─H bond strengths shown in Fig. 2, consistent with the bond energy of 22 kcal/mol for ^.^C_6_H_7_ → C_6_H_6_ + H^.^ (83). Apart from such effects, increasing the size of the hydrocarbon (or adding rings to a PAH) generally has little effect on C─H bond strength (56, 84, 85). For open-shell systems and reactive intermediates where H ejection can lead to formation of (relatively) stable, closed-shell products, greater delocalization of electron density lessens (sometimes dramatically) the cost of C─H bond cleavage (Fig. 2); in fact, within structural groups (i.e., C_9_H_9_ or C_13_H_11_), trends in decreasing C─H bond strength emerge based on the location of the bond and the aromaticity of the local environment. The hydrocarbons presented above can be roughly classified into three categories: small fuel molecules, larger radicals attributed to CHRCR processes, and closed-shell precursors to PAHs. The rate of unimolecular H ejection is strongly dependent on the strength of the C─H bonds being broken.

### Hydrogen-Ejection Rate Constants.

**Fig. 3.**
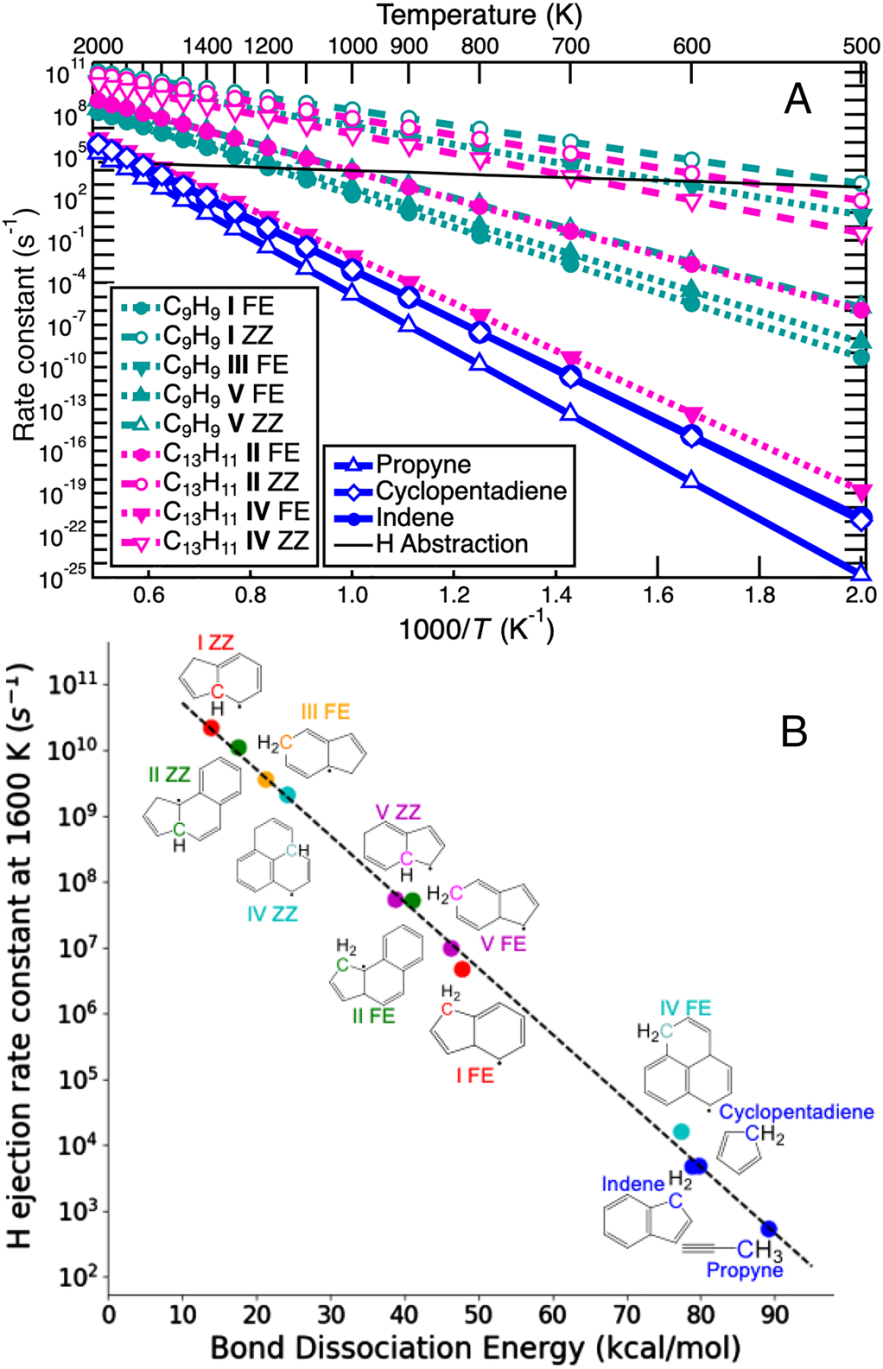
Ejection rate constants from variational transition state theory as a function of temperature and bond dissociation energy. (*A*) Canonical rate constants [k_∞_(T)] of hydrogen ejection for open- and closed-shell hydrocarbon species as a function of temperature. The rate constants for indene are difficult to distinguish from those of cyclopentadienyl. The thin black line shows an example abstraction rate constant for cyclopentadiene from Laskin and Lifschitz (87), assuming a free-H-radical concentration of 10^15^ cm^−3^ (43). Associated data are given in *SI Appendix*, Tables S2–S6. (*B*) Rate constants for hydrogen ejection are plotted on a logarithmic scale against the corresponding C─H bond dissociation energy at 0 K (ejection sites shown in color). Data are given in *SI Appendix*, Table S1.

We computed H-ejection rate constants for various species using variational transition state theory (VTST) (86). Examples of rate constants for open- and closed-shell species are shown in Fig. 3*A*. The nature of the product influences both the C─H bond strength and ejection rates. Fig. 3*B* compares 0-K C─H bond-dissociation energies (BDEs) related to ejection and ejection-rate constants at 1,600 K and demonstrates that smaller BDEs lead to larger rate constants. Indeed, the ability of a thermodynamic quantity (BDE at 0 K) to yield useful information about the VTST rate constants at 1,600 K is encouraging, as the former is much easier to compute. A summary of the values shown in Fig. 3*B* is given in *SI Appendix*, Table S1, while free energy barriers to H ejection and ejection-rate constants for these species are provided as a function of temperature in *SI Appendix*, Tables S2–S6.

We now consider the behavior for different types of hydrocarbons. C─H BDEs for many closed-shell hydrocarbons, such as cyclopentadiene (42, 88–91) and indene (92–97), have been reported previously; these bonds are relatively strong and generally hard to break with thermal energy alone. Our computed BDE for cyclopentadiene is 79.8 kcal/mol, which is in good agreement with the Active Thermochemical Tables (ATcT) (98) (81.21 ± 0.24 kcal/mol) and other previously reported values (90, 91), and our BDE for propyne of 89.2 kcal/mol is also in good agreement with the value from the ATcT (98) (90.206 ± 0.074 kcal/mol), adding confidence to the relative accuracy of the bond strengths reported here.

Propyne and cyclopentadiene therefore must both overcome high energetic barriers to break a C─H bond (Fig. 2), making H ejection from small closed-shell hydrocarbons difficult and slow even at elevated flame temperatures. Temperatures as high as 2,000 K are necessary to attain millisecond-scale ejection rates (Fig. 3), despite the formation of RSRs like propargyl and cyclopentadienyl. Larger closed-shell species, such as indene, exhibit similar ejection-rate constants compared with their smaller relatives. Fig. 3*A* shows that indene (C_9_H_8_) loses H (forming indenyl) at essentially the same rate as cyclopentadiene. Though hot spots in flames may initiate H ejection from such species, the high energetic cost of breaking this C─H bond through thermal energy alone makes such a process less likely than abstraction in most flame regions.

We have also compared our computed canonical rate constants for hydrogen ejection from indene, cyclopentadiene, and propyne with previous results provided by other groups. The comparisons are shown in *SI Appendix*, Figs. S1–S3. Our temperature-dependent rate constant for hydrogen ejection from indene [k (s^−1^) = 9.94 × 10^14^ *T*^−0.122^ exp (−40180/*T*)] agrees very well with the values published by da Silva and Bozzelli (93), as shown in *SI Appendix*, Fig. S1. Our temperature-dependent rate constant for hydrogen ejection from cyclopentadiene [k (s^−1^) = 6.07 × 10^14^ *T*^−0.0171^ exp (−40660/*T*)] demonstrates good agreement with previous results from Roy et al. (99), Kern et al. (100), Harding et al. (101), Robinson and Lindstedt (102), and Narayanaswami et al. (103), as shown in *SI Appendix*, Fig. S2. There is, however, wide variability in the results from these studies, and it is a topic worthy of further study. Our rate constant for propyne [k (s^−1^) = 9.50 × 10^14^ *T*^0.0373^ exp (−45550/*T*)] shows very good agreement with the combined experimental and theoretical results of Giri et al. (104), as demonstrated in *SI Appendix*, Fig. S3. These results are also compared with hydrogen-abstraction rate constants from previous studies in *SI Appendix*, Figs. S1–S3. The H-ejection rate constants are summarized in *SI Appendix*, Table S12, and the H-abstraction values are summarized in *SI Appendix*, Table S13.

Certain circumstances and environments may influence the efficacy of hydrogen loss from these closed-shell species. In the interstellar context, photon absorption may incur hydrogen loss (105); electronic excitation of molecules can promote reactivity and decrease C─H bond strength in some hydrocarbons as singly excited states essentially act as diradicals (106). We have found that triplet cyclopentadiene loses hydrogen easily; the free energy barrier for this process has a magnitude of around 30 kcal/mol at 1,600 K, approx. 60% less than the cost of H loss from ground-state cyclopentadiene. A similar calculation on toluene produced analogous results. This information is useful when considering hydrogen-loss processes in the ISM and in situations where molecules may have diradical character. In pyrolysis systems with residence times on the order of seconds, H-atom ejection rates to form RSRs, such as indenyl from indene or propargyl from propyne, can be large enough to initiate particle inception at relatively low temperatures despite an initial lack of hydrogen atoms available for abstraction (33, 34).

In contrast to the closed-shell molecules, open-shell species can have much faster H-ejection rates because of the formation of extensively conjugated (typically aromatic) products. The cases considered in Fig. 2 have a range for C─H BDEs of 14 to 50 kcal/mol for open-shell species, leading to the much larger H-ejection rate constants depicted in Fig. 3. An exception is ejection from the free-edge (FE) site of **IV**, which leads to a triplet product and thus involves unpairing of a C─H bonded pair of electrons much like closed-shell species. This system is unlike other open-shell systems where dissociation is accomplished by localization of a singly occupied molecular orbital on the departing H atom.

Hydrogen-ejection rates for open-shell C_9_H_9_ isomers (precursors to indene) appear to be very competitive with abstraction even at relatively low flame temperatures; such radicals lose a hydrogen atom several orders of magnitude faster than indene despite similar size and composition. As the temperature approaches 1,300 K, hydrogen ejection from the zigzag (ZZ) site of **V** [studied in (12) as an intermediate in the CHRCR mechanism] occurs on a submicrosecond timescale (*SI Appendix*, Table S4), several orders of magnitude faster than abstraction. Other isomers of C_9_H_9_ eject even faster. Hydrogen ejection occurs at submicrosecond rates at 900 K from the ZZ site with extremely low free energy barriers for both **III** (accessible via a single H migration from **V**) and **I** [an intermediate in the reaction of benzyl and C_2_H_2_ (81, 82, 107)], as shown in Fig. 3.

As demonstrated for **I** and **V** in Fig. 3, there is considerable site sensitivity to ejection-rate constants, which are influenced by sterics and (de)aromatization. Loss of ZZ hydrogen from C_13_H_11_ isomers occurs readily on the order of microseconds at relatively low temperatures (800 to 1,000 K) as this H loss results in aromatizing rings. ZZ ejection from **IV** results in an increase in aromaticity from a single benzene ring to a two-ring aromatic structure. Similarly, the very rapid ejection from the ZZ site of **II** results in the formation of a two-ring aromatic structure. By contrast, ejection from FE sites on many of these molecules leads to less extensive conjugation and is hence markedly slower, as shown in Fig. 3, with ejection from the FE site of **IV** being almost as energetically costly as H ejection from closed-shell indene or cyclopentadiene (as previously discussed). On the other hand, ejection from the FE site in the six-membered ring in **III** results in the formation of a benzene ring and is thus quite energetically favored. Although C─H bond strengths are generally expected to be weaker for tertiary (ZZ) than for secondary (FE) carbons for a given molecule, the degree of resulting conjugation/aromatization also has an important impact on ejection-rate constants.

Abstraction of hydrogen atoms by free radicals is the main competitive process to hydrogen ejection. Previously published abstraction rate constants for cyclopentadiene provide values between 2.6 × 10^4^ and 2.6 × 10^5^ s^−1^ at 2,000 K, assuming 10^15^ cm^−3^ (43) for typical free H-radical concentrations in flames. An example from Laskin and Lifschitz (87) is shown in Fig. 3*A*, and other values are presented in *SI Appendix*, Fig. S2. Actual abstraction rates will vary with hydrocarbon concentrations and variations in H partial pressure (108–112). As shown in Fig. 3, H abstraction by free radicals is much more likely for stable closed-shell hydrocarbons than H ejection at typical flame temperatures. On the other hand, at temperatures above 1,200 K, ejection rates for most reactive intermediates are easily greater than hydrogen abstraction. Dehydrogenation via abstraction is an appealing route because of the extremely low free energy barriers (<20 kcal/mol), which makes for extremely fast rates of hydrogen loss; however, there are many factors that decrease the effectiveness of these processes. Abstraction events require collision of the abstractor and target molecule, where the collision frequency is linearly dependent on radical concentration but has only a $\sqrt{T}$ dependence on temperature. A high enough abstractor concentration is necessary to produce an abstraction-dominated environment, as radicals are generally highly reactive, and a very high concentration would lead to pairwise radical annihilation. While the primary reaction zones of combustion systems generally have a persistently high free radical equilibrium, such high free radical concentrations are not achievable for all relevant environments in which hydrocarbon growth and particle inception may occur. Some environments, such as the postflame zone in combustion, during pyrolysis, and in the ISM, simply cannot maintain high enough small-radical concentrations to promote an abstraction-dominated environment, indicating that other processes, such as H ejection, may be very important for propagating reactions.

To this point, we have demonstrated that canonical hydrogen-ejection rates from open-shell hydrocarbons occur rapidly at a range of temperatures relevant for combustion and pyrolysis and that these rates may be much faster than competing dehydrogenation processes, such as abstraction. While ejection is already fast from some sites on isomers **I** to **V** at thermal equilibrium (Fig. 3), it is likely to be faster still under combustion and pyrolysis conditions, as discussed below.

### Hydrogen Ejection and the First Steps of CHRCR.

A large focus of this paper is to explore hydrogen ejection as a viable submechanism of the CHRCR or similar pathways; H ejection facilitates molecular-weight growth and drives hydrocarbon clustering while regenerating radical species and producing new local abstractors. To put our findings into better context, we have explored an early reaction sequence of the CHRCR pathway involving C_9_H_9_ isomers included in our study.

**Fig. 4.**
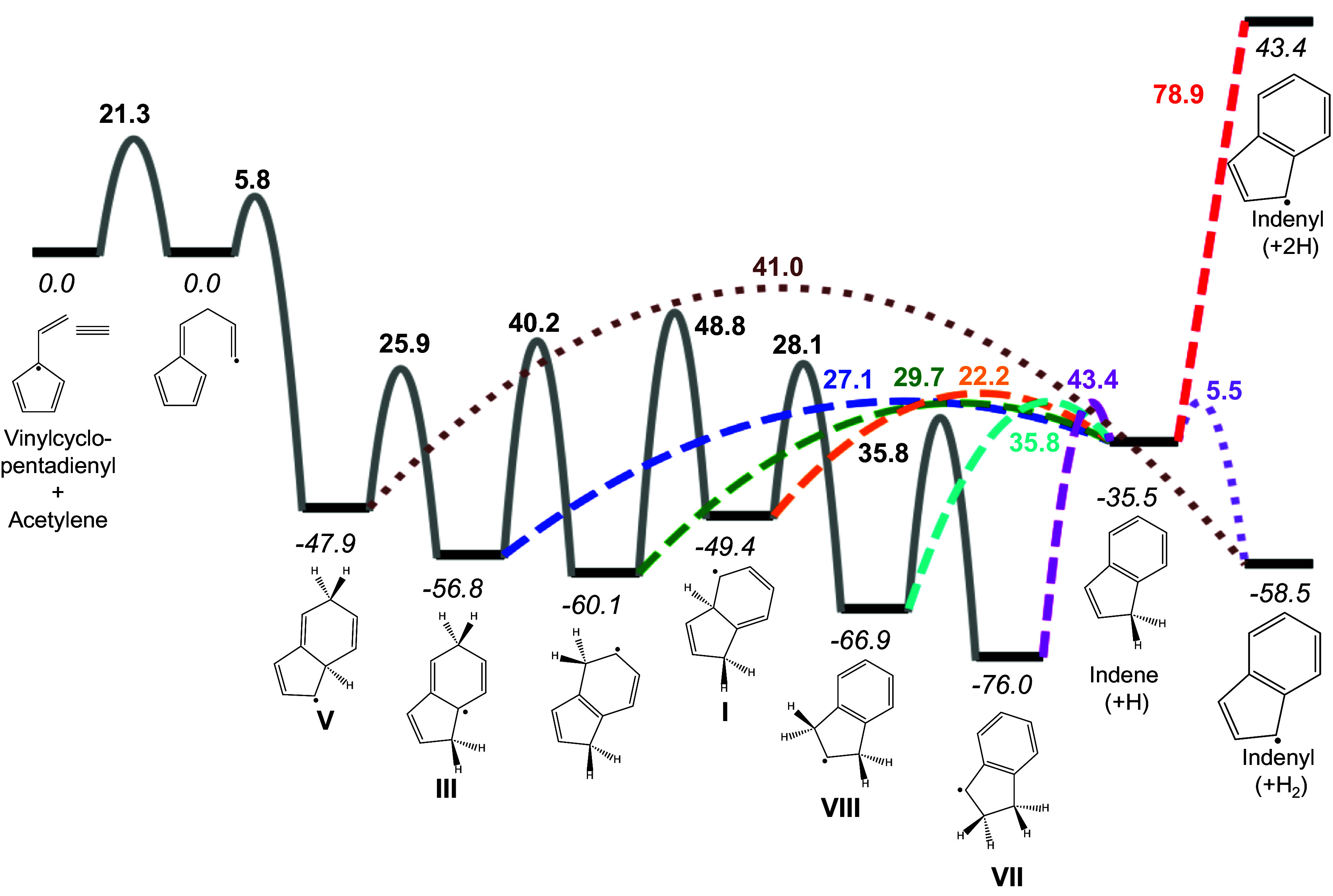
Potential energy surface (PES) for the sample CHRCR reaction sequence from vinylcyclopentadienyl + acetylene to indenyl. Energies of the minimum energy structures (*italicized*) relative to the van der Waals complex between vinylcyclopentadienyl + acetylene are shown in kcal/mol, as are energetic barriers for accessing transition state structures from the preceding minima (**bolded**) for both isomerization (black) and H-ejection processes (colored). The energies shown here correspond to 0 K (i.e., only changes in electronic and zero-point vibrational energies are considered). The C_9_H_9_ isomer **V** is initially formed via ring closure, and various pathways to indene are possible via isomerization followed by H ejection. From nearly all C_9_H_9_ isomers explored here, ejection, as a primary route of hydrogen loss, is rapid, as shown in Table 1. Subsequent H ejection to form indenyl and 2 H^.^ is barrierless in the electronic energy and involves a direct 78.9 kcal/mol uphill climb in the zero-point corrected energy. However, the first ejected H^.^ atom can act as an abstractor and lead to formation of indenyl + H_2_ with only a 5.5 kcal/mol barrier. This pathway and a concerted H_2_ ejection from V leading to the same products are shown with dotted colored lines.

Fig. 4 shows the potential energy surface (PES) for an example cyclization reaction between acetylene and vinylcyclopentadienyl producing **V** and various reaction steps involving H ejection. Vinylcyclopentadienyl was identified in the experiments of Michelsen and coworkers (12, 53, 113) as an RSR that may play an important role in the CHRCR pathway. Theoretical studies by Mao et al. (65) and Martí et al. (113) suggest that vinylcyclopentadienyl is predominantly produced during the reaction of cyclopentadienyl with acetylene. Mao et al. (65), however, concluded that tropyl (i.e., cycloheptatrienyl) is the major isomeric product of this reaction at flame temperatures and that vinylcyclopentadienyl and benzyl are minor C_7_H_7_ products. An experimental study by Savee et al. (82) confirms that tropyl is the only major product at temperatures below 1,000 K. The results of Martí et al. (113) indicate that vinylcyclopentadienyl and benzyl dominate at higher temperatures, which is consistent with the experimental results of Rundel et al. (53). In addition, Martí et al. (113) and Meng et al. (114) discovered a previously unknown pathway between vinylcyclopentadienyl and benzyl, suggesting that vinylcyclopentadienyl is an important intermediate between cyclopentadienyl + acetylene and benzyl.

Indene has been demonstrated experimentally and theoretically to be produced from the reactions of acetylene with the C_7_H_7_ isomers tropyl (65, 82) and benzyl (65, 107, 115, 116). Mao et al. (65) also performed a theoretical study of indene production from the reaction of the third important C_7_H_7_ isomer, vinylcyclopentadienyl, with acetylene. The relative energies of the intermediates and transition states reported in Fig. 4 compare within a few kcal/mol with corresponding values from Mao et al. (65) for this reaction, as shown in *SI Appendix*, Table S14.

The initial product of cyclization in the reaction of acetylene with vinylcyclopentadienyl is the C_9_H_9_ isomer **V**, for which several decomposition pathways are available. The most plausible scenarios for **V** are 1) dehydrogenate via collision with an abstractor, 2) fall apart into smaller hydrocarbons, 3) eject a hydrogen atom to form a closed-shell species, or 4) isomerize via H migration to a lower energy C_9_H_9_ isomer.

Although dehydrogenation via collision with an abstractor is a viable mechanism, collisions of **V** with small abstractors such as H^.^ occur on the order of 10^6^ s^−1^, which is dependent on the density of abstractors and scales only as $\sqrt{T}$ with temperature. H ejection, on the other hand, becomes exponentially faster with increasing temperature, and under combustion and pyrolysis conditions is a more rapid dehydrogenation route for many species (including **V**). The second mechanism listed above, decomposition into smaller hydrocarbon species, is unlikely. The newly formed C─C bonds of **V** during ring closure are strong, and breaking these species immediately into smaller fragments is thermodynamically unfavorable. Ejection and isomerization are thus left as the most plausible decomposition routes for **V**.

The canonical rate constant for ejection from the ZZ site of **V** is 5.4 × 10^7^ s^−1^ at 1,600 K. This rate constant is large, but the actual rate constant for H ejection is likely larger. To determine the behavior of **V** in the reaction context, we have computed microcanonical rate constants to account for the effects of internal energy gained via C─C bond formation (approx. 48 kcal/mol from ring closure), as well as the contribution from the thermal vibrational energy of each reactant (approx. 70 kcal/mol, assuming an initial temperature of 1,600 K). Accounting for this excess energy, the ZZ-site rate constant for ejection from **V** increases to 5.6 × 10^8^ s^−1^, an order of magnitude greater than the canonical rate constant.

Given the great amount of internal energy **V** has accumulated after ring closure, rapid isomerization to a more stable isomer is also viable (and indeed, likely). As shown in Fig. 4, several lower-lying C_9_H_9_ isomers are available (**I**, **III**, **VII**, and **VIII**). Canonical H-ejection rate constants are already large for some of these species, and excess internal energy from the preceding reaction scheme speeds hydrogen loss to an even greater degree, as shown in Table 1. Internal energy provides at least an order of magnitude boost to hydrogen-ejection rates for **V** and other C_9_H_9_ isomers. Consider a likely sequence from vinylcyclopentadienyl → **V** → **VII** and so on. **V** is initially formed with approx. 48 kcal/mol excess energy from the reaction and can isomerize to the more stable isomer **III** with a single H-atom migration, which has an even larger computed microcanonical H-ejection rate constant of 2.8 × 10^10^ s^−1^. **III** could potentially also undergo multistep isomerization to the most stable isomer **VII**, which lies almost 30 kcal/mol lower in energy than the initially formed **V** isomer. H ejection from **VII** nonetheless occurs with a rate constant of 1.4 × 10^9^ s^−1^. These rapid microcanonical rates show that, even after reorganization to lower-lying isomers, H ejection from C_9_H_9_ is much faster than other hydrogen-loss methods, such as abstraction.

**Table 1.** Canonical and microcanonical hydrogen-ejection rates for indene formation from some of the C_9_H_9_ species shown in Fig. 4

Species	Canonical ejection rate	Microcanonical ejection rate
**V** (ZZ site)	5.4 × 10^7^ s^−1^	5.6 × 10^8^ s^−1^
**I** (ZZ site)	2.2 × 10^10^ s^−1^	8.6 × 10^10^ s^−1^
**III** (FE site)	3.6 × 10^9^ s^−1^	2.8 × 10^10^ s^−1^
**VIII** (FE site)	2.3 × 10^8^ s^−1^	4.7 × 10^9^ s^−1^
**VII** (FE site)	2.8 × 10^7^ s^−1^	1.4 × 10^8^ s^−1^

Canonical rates are computed at 1,600 K; microcanonical rates assume reactants (vinylcyclopentadiene and acetylene) at a temperature of 1,600 K.

The rate of H ejection generalizes well to similar reaction schemes. Benzyl is an important C_7_H_7_ isomer in combustion and pyrolysis (12, 34, 53, 113, 117) and is a precursor to the initial C_9_H_9_ intermediate **I** from which H ejection may occur. Considering the C─C bond formation energy and that which is available vibrationally from benzyl and acetylene, the rate constant for H ejection from **I** is 3.6 × 10^10^ s^−1^. This evidence for the viability of H ejection suggests that it could be relevant for pathways involving other RSRs, not just following reactions of vinylcyclopentadienyl.

The microcanonical rate for H ejection from **V** and other C_9_H_9_ isomers is rapid, and few factors will be influential enough to significantly slow these processes. Dissipation of energy through collisions with inert gas molecules or through a natural dispersal of energy in vibrational modes may inhibit the efficiency of hydrogen ejection, but even these processes should not change the rate to a large degree. Considering a generous cross-sectional impact area for C_9_H_9_ with a diameter of several C─H bonds, collisions with an inert gas, such as N_2_, occur on the order of 10^9^ s^−1^ at a pressure of 1 bar. The effective temperature of **V** is calculated to decrease by less than 15 K per collision (assuming highly efficient energy transfer); thus, it would take at least 10 collisions to have a significant decrease in energy. Therefore, we are confident that the computed microcanonical rate constants provide a good estimate for immediate H ejection, which will take place before collisions can dissipate excess internal energy.

Species such as C_9_H_9_ are small enough that, even in the event of full internal vibrational relaxation (IVR), the internal energy gained from C─C bond-formation processes should facilitate H-ejection processes, as shown by the microcanonical rate constants described above being larger than the 1,600 K VTST canonical rate constants. However, the difference between microcanonical and canonical rate constants will decrease with increasing hydrocarbon size as IVR distributes the energy released by C─C bond formation over many more vibrational modes.

Hydrogen ejection is a rapid process that is very likely to occur along the CHRCR reaction pathways. However, the efficacy of the second hydrogen ejection (as shown in Fig. 4, where closed-shell indene loses a hydrogen to become indenyl) is less certain. Figs. 2 and 3 demonstrate that H ejection for closed-shell species is slow because of higher C─H bond strengths. Indene, for example, has a bond-dissociation energy of nearly 80 kcal/mol. Even with significant thermal energy from the flame, hydrogen ejection is unlikely to be as competitive with abstraction for indene as for the C_9_H_9_ species. In areas where abstractor concentrations are high, collision with an abstractor seems more likely than spontaneous hydrogen ejection for a closed-shell species. Hydrogen ejection may of course still occur from indene (albeit not as quickly as for C_9_H_9_), and this may be important in low-pressure regions to propagate reaction sequences leading to the next radical clustering center. In this way, ejection and abstraction processes are likely to both be at play in the CHRCR mechanism, depending on the conditions under which the reaction sequence is taking place. Abstraction itself may be initiated and sped up by ejection events in a pseudounimolecular manner, as discussed in the next section.

### Sequential H Ejection and Processes of Larger PAHs.

Hydrogen ejection from an open-shell species results in a closed-shell molecule, which needs to lose another H atom to reform a radical. Loss of both hydrogen atoms enables chain-reaction propagation and soot formation by the CHRCR mechanism (12). In environments where small radical abstractors are scarce, sequential ejections from a single molecule may be vital for continuation of the chain reaction.

**Fig. 5.**
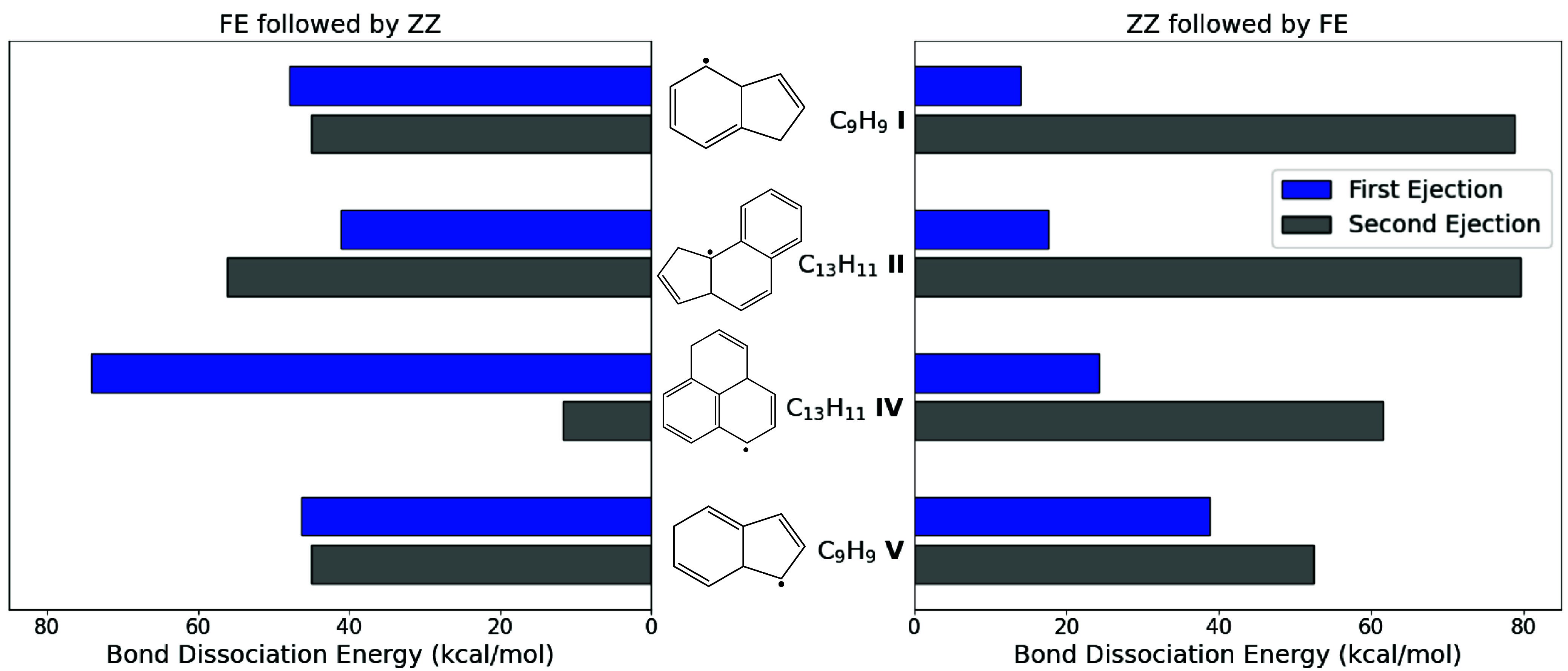
Bond-dissociation energies (electronic and zero-point vibrational energy changes at 0 K) for sequential H ejection from isomers **I, II**, **IV**, and **V**. Significantly lower C─H bond energies at ZZ sites make ejection from such sites the preferred pathway, and the second ejection is typically more energetically demanding. Ejection of H_2_ (as opposed to 2H^•^ is energetically favorable for all four species, by −9.1 kcal/mol (**I**), −4.6 kcal/mol (**II**), −16.1 kcal/mol (**IV**), and −10.6 kcal/mol (**V**). As a result, the loss of the second H atom following initial ZZ ejection is likely to be an abstraction process by either the first ejected H atom (via a concerted transition state or roaming reaction) in low-density environments like the ISM, or by other radicals in general in flame environments.

Sequential hydrogen loss from either C_13_H_11_ or C_9_H_9_ isomers results in a new RSR (via a closed-shell intermediate) and two H atoms. Fig. 5 demonstrates the differences in bond-dissociation energies between the first and second H ejections from isomers **I, II**, **IV**, and **V**. As seen previously, initial ZZ ejections are easier than those from FE sites, indicating that this route will be the preferred ejection pathway. A second hydrogen atom ejection from the FE site of closed-shell C_9_H_8_ and C_13_H_10_ isomers resulting from an initial ZZ ejection is more energetically expensive. On the other hand, subsequent ZZ ejection from the C_9_H_8_ and C_13_H_10_ products of FE ejection from **I, IV,** and **V** is energetically more favorable than the first FE ejection.

These effects are due to the molecular environment; initial H ejections that lead to stable aromatic rings have low barriers, and thus occur quickly, as depicted in Fig. 5. This stability-driven site preference leads to the high energetic cost for the second ejection. For example, initial ejection from the ZZ site of **IV** is fast, with a bond dissociation energy of only about 24 kcal/mol, resulting in closed-shell C_13_H_10_ (**IV**-H) and leading to two aromatic rings. The second ejection to form C_13_H_9_ is more than twice as costly. While this open-shell product benefits from many resonance structures, hydrogen loss does not stabilize this molecule relative to the parent, as aromatization of all three six-membered rings is not feasible. This logic may be extended to sequential ejection from **II** and explains why initial FE ejections are so difficult. Interestingly, **V** has relatively similar barriers for the first and second ejections, with the latter being only slightly more costly regardless of initial ejection site. This similarity may result from competition between the favorable aromatization of the six-membered ring and loss of some closed-shell stability.

Sequential H ejection thus appears difficult as a stand-alone process in many cases from an energetic standpoint, particularly for high-temperature and pressure combustion events. Large bond-dissociation energies in the closed-shell species lead to slower rates of H ejection. In instances where abstractor concentrations are adequately high, reaction propagation in the CHRCR context will likely require an abstraction event to lose the second hydrogen atom.

In some cases, multiple dehydrogenations via H ejection may be more viable. One scenario where sequential H ejection may be a more useful pathway is in the postflame region of combustion flames and during pyrolysis. Soot formation occurs under these conditions, despite low abstractor abundances, requiring pathways that proceed without the need for abstraction (11). Despite the slow speed of the second ejection, it may still occur faster than abstraction in this case, since abstraction processes depend directly on the concentrations of small radicals, which determine the likelihood of collision. Another similar scenario is in the ISM, where collisions will be even rarer for most systems. And finally, the success of the first ejection may promote an abstraction on the same molecule; the first ejection creates an H radical in close proximity to the parent molecule, which may abstract a second hydrogen atom in the style of a roaming-atom reaction (118–120). This sequence would effectively result in H_2_ loss without any change in the overall radical concentration, with far more favorable energetics (Fig. 5). Concerted H_2_ loss via a single transition state is possible from species such as **V**, with 0 K barrier heights similar to those for cleaving the weakest C─H bond (approx. 40 kcal/mol). Coordinates for the structure of this transition state is given in the Zenodo repository (121) associated with the *SI Appendix*.

## Conclusions

The data presented in this paper highlight several important concepts. We have tabulated C─H bond strengths for a variety of open- and closed-shell hydrocarbons and found a striking range of values that depend strongly on the species and the structural characteristics. We have observed that C─H bond strengths for normal valent, closed-shell molecules have high bond-dissociation energies, which typically fall into the expected range. In sharp contrast, open-shell species (even with similar structural characteristics) tend to have much smaller bond-dissociation energies, as low as below 30 kcal/mol in some cases.

This range of C─H bond strengths directly affects hydrogen-ejection rates. Our results show that the significance of hydrogen ejection depends strongly on the nature of the hydrocarbon species and other factors, such as the temperature and concentrations of hydrogen abstractors. These data indicate that H ejection is energetically costly and rather slow for most closed-shell species, but open-shell species, particularly those thought to be intermediates in CHRCR growth pathways, can eject hydrogen atoms rapidly in a site-specific manner, with rates of H loss from zigzag sites outstripping those from free-edge sites. The high ejection rates from *sp*^3^ sites in multiring radical species are particularly noteworthy, as PAHs are widely thought to be key precursors to incipient soot particles (11). Rates of H ejection and the associated C─H bond strengths are heavily influenced by general factors such as the gain (or loss) of aromaticity in molecular rings, suggesting that families of similarly structured hydrocarbons should exhibit predictable trends in ejection rates (106).

Our results indicate that small bond-dissociation energies in PAH radicals contribute to fast hydrogen-ejection rates. This process can occur rapidly at combustion and pyrolysis temperatures and potentially help propagate reaction pathways. Since hydrogen loss from closed-shell species for a given reaction sequence is comparatively slow, we theorize that hydrogen ejection and hydrogen abstraction are both critical processes acting to support CHRCR pathways.

Hydrogen ejection may be a key process in radical-driven mechanisms acting in several stages of these soot-growth pathways. Prior to and during inception, loss of H atoms by ejection from thermally excited or postcyclization precursors and intermediates allows for rapid growth of PAHs and RSRs, which in turn facilitates fast intermolecular reaction rates and speeds radical-chain reactions. Characterization of a CHRCR reaction sequence in this work modeled the relevance of dehydrogenation of intermediates via ejection. During the inception event, stabilization of dimers and larger structures (such as the clusters at radical growth centers proposed by CHRCR) following covalent bond formation may occur more quickly via hot-hydrogen ejection (12) and negate the need for collisional stabilization. H ejection may also play a role in later stages of soot formation, where new mechanisms are emerging that promote soot mass growth in the absence of high H-atom concentrations; for example, fast hydrogen loss from the surfaces of incipient and partially aged particles creates radical sites where three-dimensional growth occurs to eventually form the primary-particle core-shell structure and complex aggregate of mature soot.

Rapid rates of hydrogen loss for open-shell intermediates are also relevant for PAH-formation processes in the ISM, either in hot or cold regions, where abstractors and energetic collisions are scarce. Under conditions where hydrogen ejection is a preferred pathway acting in radical-chain reactions, the radicals are regenerated. This process may also help explain the prevalence of particle inception and growth in the secondary reaction zone of flames (and perhaps in environments such as cold molecular clouds or planetary atmospheres), where radicals are depleted and activation of a hydrocarbon by H abstraction is far less likely (11).

The data presented here provide numerical evidence that hydrogen ejection is a significant component of radical-driven soot inception, sometimes acting alongside hydrogen abstraction; this process may contribute to multiple stages of soot formation and growth and even to radical-driven processes that act in the formation of large interstellar PAHs. Our results demonstrate the need for further investigation to systematically explore the role of hydrogen ejection in chemical kinetic models containing radical-chain reactions contributing to inception and growth of soot particles. Future work will involve performing calculations of temperature- and pressure-dependent rate constants using a master-equation method, e.g., (41, 49, 107, 122).

## Materials and Methods

All electronic structure calculations were performed in the methodological framework of Kohn–Sham density functional theory (DFT) using the Q-Chem software package (123). Geometry optimizations and harmonic vibrational frequencies were obtained using the ωB97M-V functional (124, 125), a range-separated hybrid meta-GGA functional that is the most accurate of its class for main group chemistry (126, 127), and the def2-SVPD basis set (128, 129). Single-point energies at the optimized geometries were subsequently computed with ωB97M-V and the larger def2-TZVPD basis set. All local exchange-correlation integrals were evaluated on an ultrafine integration grid consisting of 99 radial points and 590 angular Lebedev points. The BDEs reported in this work were computed from electronic energies corrected with zero-point vibrational energies (without any scaling of vibrational frequencies) and therefore correspond to a temperature of 0 K. The transition states shown in Fig. 4 are first-order saddle points in the electronic energy alone vs nuclear displacements, and barrier heights reported in that figure were corrected for zero-point vibrational energies. The corresponding saddle-point structures were found with the use of the freezing-string method (130) to obtain initial structures, followed by optimization of the resulting saddle point using the partitioned-rational function optimization eigenvector following method (131) and verification via a frequency calculation. The transition state for concerted H_2_ loss from **V** was similarly obtained.

Thermal (canonical) rate constants for H ejection were computed using variational transition state theory (132) along the C─H dissociation path. Constrained optimizations for a range of C─H distances along the dissociation path were carried out with ωB97M-V/def2-SVPD. Free energies corresponding to these geometries were found by combining the electronic energy at the ωB97M-V/def2-TZVPD level with a ωB97M-V/def2-SVPD nuclear free energy computed from a modified quasi-rigid-rotor harmonic-oscillator model (RRHO) with a cutoff of 100 cm^−1^ to avoid issues associated with low-frequency vibrational modes (133). The unimolecular nature of the process leads to no change in the nuclear free energy associated with translational degrees of freedom and a lack of pressure dependence. The geometry with the highest free energy at a given temperature (ranging between 500 to 2000 K, in increments of 100 K) was selected as the transition state, and the rate for H ejection (as shown in Fig. 3) was computed with the Eyring equation using the free energy difference between this geometry and the corresponding minimum energy structure. The resulting rates are reported in *SI Appendix*, Tables S2–S7. We validated the accuracy of our approach through comparison with results for H ejection from cyclopentadiene and the ZZ site of species **I** using the more accurate ωB97M (2) double hybrid functional (134) and the aug-cc-pVTZ basis for calculating electronic energies (using the geometry and nuclear free energies obtained from ωB97M-V/def2-SVPD). These results are reported in *SI Appendix*, Tables S8 and S9, showing good agreement with the pure ωB97M-V based approach employed in this work.

Microcanonical (constant energy) rate constants for H ejection from several C_9_H_9_ species were computed with RRKM theory (132), using the Beyer-Swinehart algorithm (135), for computing the vibrational density of states at a given energy. All frequencies below a cutoff (here, 100 cm^−1^) were replaced by the cutoff value. The values reported in Table 1 were found from computing the average rate $\left\langle k\right\rangle =\int k\left(E\right)p\left(E\right)dE$ over the energies E available to the system from the electronic and vibrational energy of the reactants vinylcyclopentadienyl and acetylene. The corresponding probability distribution $p\left(E\right)$ was constructed from quantum Boltzmann sampling of the vibrational degrees of freedom at 1,600 K, within the harmonic oscillator approximation and applying the low-frequency cutoff. An alternative approach is to use RRKM rates $k\left(\left\langle E\right\rangle \right)$ arising from the average reactant energy $\left\langle E\right\rangle =\int Ep\left(E\right)dE$, which are reported in *SI Appendix*, Table S10 for acetylene + C_7_H_7_ reactions and are generally slightly smaller. The effect of a different cutoff frequency (500 cm^−1^) on the microcanonical rates is also reported in *SI Appendix*, Table S10, and is generally found to lead to an increase in the H ejection rate. We also report in *SI Appendix*, Table S11 microcanonical rates arising from C_9_H_9_ species formed via benzyl reacting with acetylene at 1,600 K, which are smaller because benzyl is lower in energy than vinylcyclopentadienyl, but still larger than the canonical 1,600 K rates.

## Supplementary Material

Appendix 01 (PDF)

Dataset S01 (XLSX)

## Data Availability

Electronic structure inputs and outputs, scripts for rate constant evaluation, and raw data have been deposited in a Zenodo repository (121). All other data associated with this study are included in the article and/or supporting information.
