# Supplementary material for: Hydrogen ejection from hydrocarbons: Characterization and relevance in soot formation and interstellar chemistry
Source: Proc Natl Acad Sci U S A. 2024 Dec 9;121(51):e2202744121. doi: 10.1073/pnas.2202744121 (PMC11665904; doi:10.1073/pnas.2202744121)
Supplement: Supplementary file 1 — Appendix 01 (PDF) [file pnas.2202744121.sapp.pdf]

PNAS

Supporting Information for

Hydrogen ejection from hydrocarbons: Characterization and relevance in soot formation and interstellar chemistry.

Josie Hendrix,[a,1] Diptarka Hait,[*,a,b,c,1] Hope A. Michelsen,[*,d] and Martin Head-Gordon[*,a,b]

[a]Department of Chemistry, University of California, Berkeley, CA, USA 94720

[b]Chemical Sciences Division, Lawrence Berkeley National Laboratory, Berkeley, CA, USA 94720

[c]Department of Chemistry and The PULSE Institute, Stanford University, Stanford, CA, USA 94305

[d]Department of Mechanical Engineering and Environmental Engineering Program, University of Colorado, Boulder, CO, USA 80309

*Diptarka Hait, Hope A. Michelsen, Martin Head-Gordon

Email: diptarka@berkeley.edu; hope.michelsen@colorado.edu; mhg@cchem.berkeley.edu

[1]These authors contributed equally to this paper.

This PDF file includes:

Figure S1 to S3
Tables S1 to S14
SI References

Other supporting materials for this manuscript include the following:

Dataset S1
Zenodo repository: https://doi.org/10.5281/zenodo.13307485

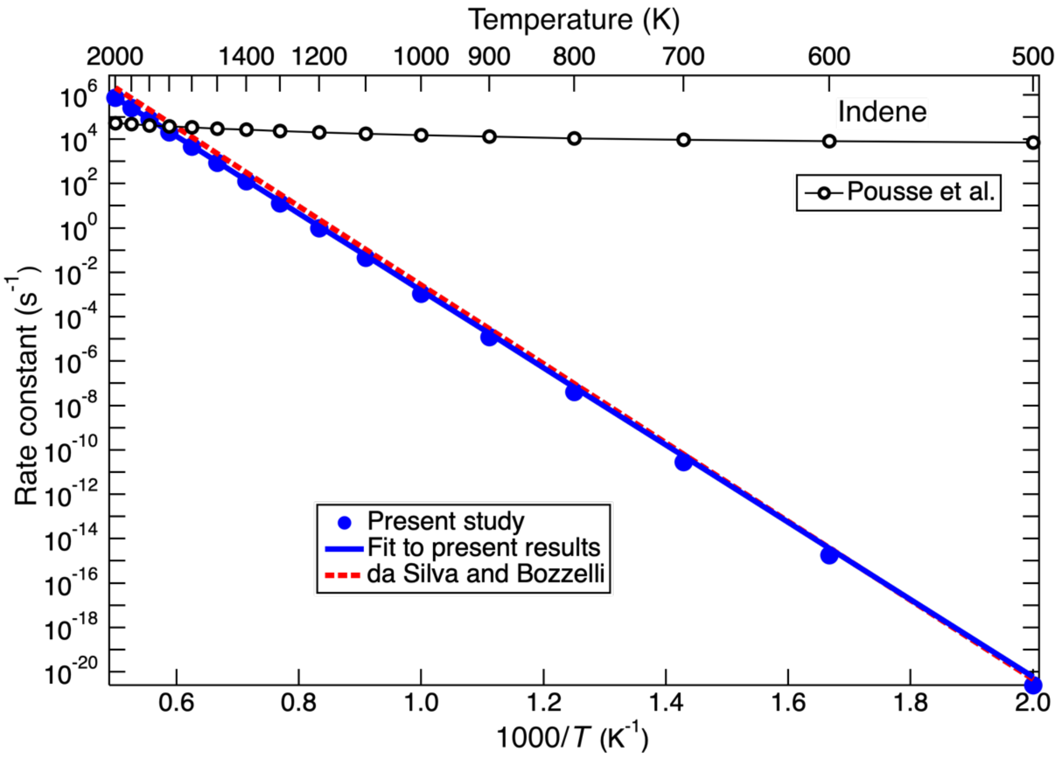

Figure S1. H-ejection and H-abstraction rate constants as a function of temperature for indene. Solid blue circles represent calculated H-ejection rate constants from the present study; values are provided in Table S6. The solid blue line shows the fit to the calculated rate constants. The red dashed line shows H-ejection rate constants from da Silva and Bozzelli (1). The open black circles and black line represent the rate constants for H-abstraction from indene by H atoms from Pousse et al. (2), assuming an H-atom concentration of $1x10^{15}$ cm^{-3} (3) for typical free-H-radical concentrations in flames. Arrhenius parameters for the H-ejection rate constants are given in Table S12, and those for the H-abstraction rate constants are given in Table S13.

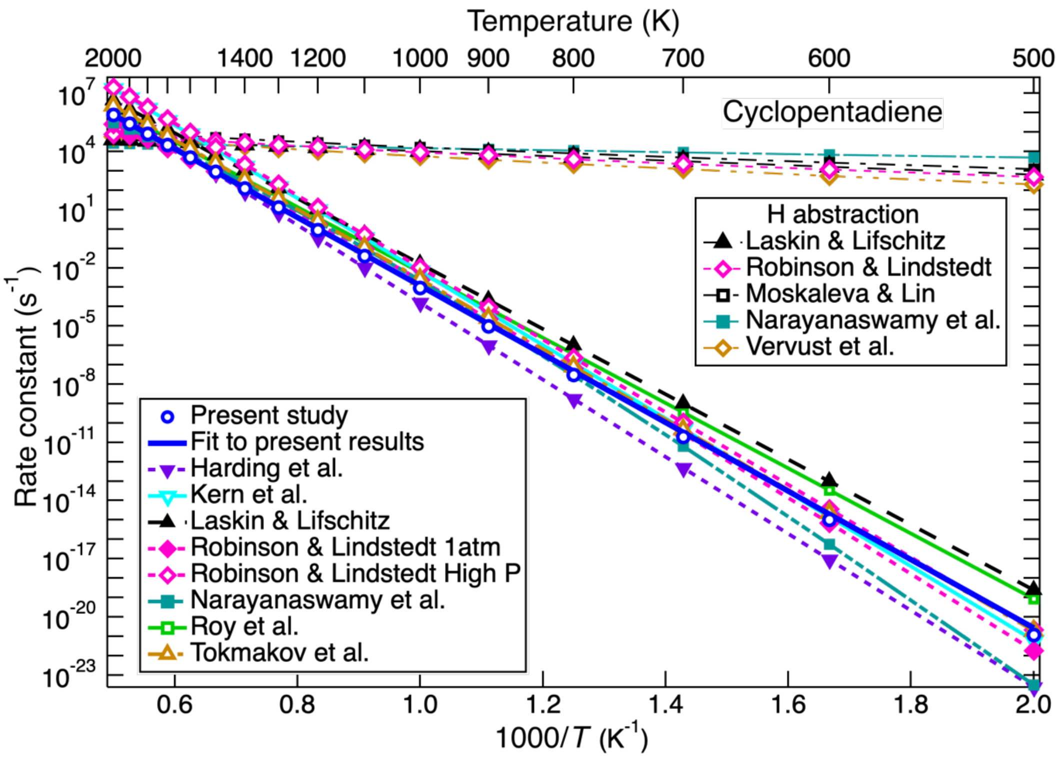

Figure S2. H-ejection and H-abstraction rate constants as a function of temperature for cyclopentadiene. Open blue circles represent calculated H-ejection rate constants from the present study; values are provided in Table S6. The solid blue line shows the fit to the calculated rate constants. The other thick lines show H-ejection rate constants from Harding et al. (4) [as given by Robinson and Lindstedt (5)], Kern et al. (6), Laskin and Lifschitz (7), Robinson and Lindstedt (5), Narayanaswamy et al. (8), Roy et al. (9), and Tokmakov et al. (10), as indicated in the legend. The thin lines represent the rate constants for H-abstraction from cyclopentadiene by H atom from Laskin and Lifschitz (7), Robinson and Lindstedt (5), Moskaleva and Lin (11), Naranaswamy et al. (8), and Vervust et al. (12), as indicated in the upper right legend. The H-atom concentration was assumed to be 1×10^{15} cm^{-3} (3) typical of free-H-radical concentrations in flames. Arrhenius parameters for the H-ejection rate constants are given in Table S12, and those for the H-abstraction rate constants are given in Table S13.

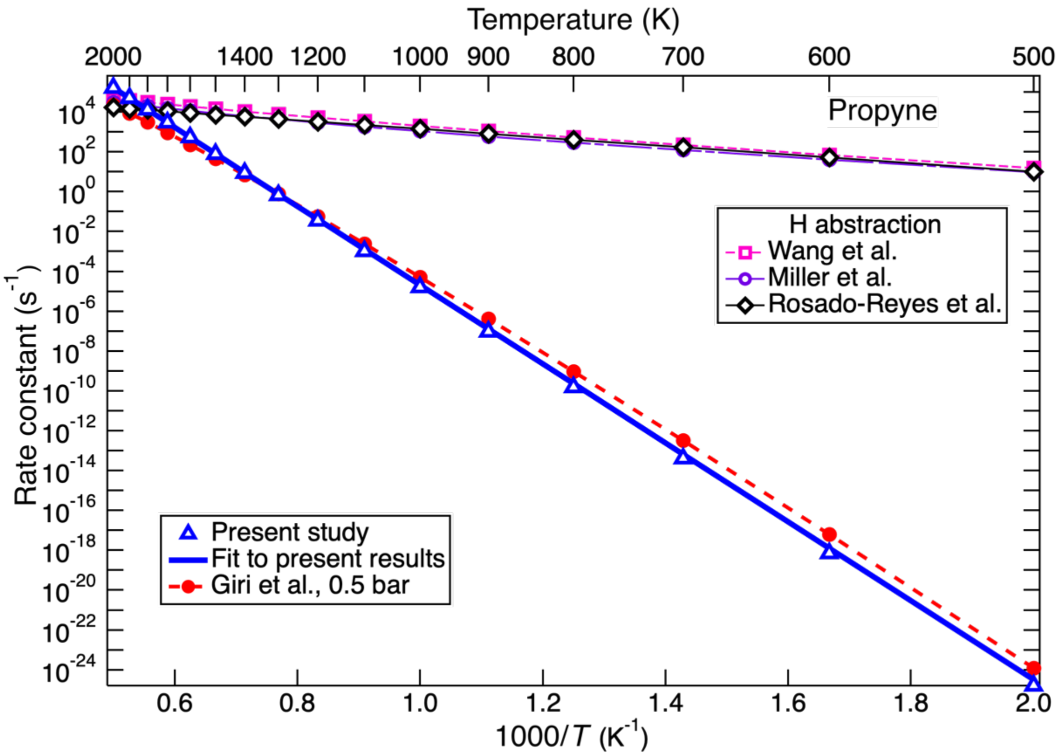

Figure S3. H-ejection and H-abstraction rate constants as a function of temperature for propyne. Open blue triangles represent calculated H-ejection rate constants from the present study; values are provided in Table S6. The solid blue line shows the fit to the calculated rate constants. The red thick line shows H-ejection rate constants from Giri et al. (13), as indicated in the legend. The thin lines represent the rate constants for H-abstraction from propyne by H atom from Wang et al. (14), Miller et al. (15), and Rosado-Reyes et al. (16), as indicated in the upper right legend. The H-atom concentration was assumed to be $1x10^{15}$ cm^{-3} (3) typical of free-H-radical concentrations in flames. Arrhenius parameters for the H-ejection rate constants are given in Table S12, and those for the H-abstraction rate constants are given in Table S13.

Table S1. C-H bond-dissociation energies (BDE, 0 K), variational transition-state theory free energy barriers to H ejection ($\Delta A^{\ddagger}$, 1600 K) and corresponding H-ejection rate constants (from Eyring equation) for species in Fig. 3b.

Species	BDE (kcal/mol) 0 K	$\Delta A^{\ddagger}$ (kcal/mol) 1600 K	Rate constant (s^{-1}) 1600 K
I (ZZ)	13.9	23.3	2.2E+10
II (ZZ)	17.6	25.5	1.1E+10
III (FE)	21.3	29.0	3.6E+09
IV (ZZ)	24.2	30.8	2.1E+09
V (ZZ)	38.8	42.4	5.4E+07
II (FE)	41.1	42.5	5.2E+07
V (FE)	46.3	47.8	9.8E+06
I (FE)	47.8	50.2	4.7E+06
IV (FE)	77.4	68.2	1.6E+04
Indene	78.9	72.1	4.7E+03
Cyclopentadiene	79.8	72.0	4.8E+03
Propyne	89.2	79.1	5.3E+02

Table S2. Free-energy barriers to H ejection ($\Delta A^{\ddagger}$) from variational transition-state theory and corresponding H-ejection rate constants (from Eyring equation) at various temperatures for $C_{13}H_{11}$ (**II**) from the ZZ and FE sites.

	ZZ ejection		FE ejection	
Temperature (K)	$\Delta A^{\ddagger}$ (kcal/mol)	Rate const. (s^{-1})	$\Delta A^{\ddagger}$ (kcal/mol)	Rate const. (s^{-1})
500	25.5	7.4E+01	43.4	1.1E-06
600	25.5	6.4E+03	43.3	2.1E-03
700	25.5	1.6E+05	43.2	4.9E-01
800	25.5	1.8E+06	43.1	2.8E+01
900	25.5	1.2E+07	43.0	6.9E+02
1000	25.5	5.6E+07	42.9	8.9E+03
1100	25.5	2.0E+08	42.8	7.3E+04
1200	25.5	5.7E+08	42.7	4.2E+05
1300	25.5	1.4E+09	42.6	1.9E+06
1400	25.5	3.1E+09	42.6	6.6E+06
1500	25.5	6.1E+09	42.5	2.0E+07
1600	25.5	1.1E+10	42.5	5.2E+07
1700	25.5	1.9E+10	42.5	1.2E+08
1800	25.5	3.0E+10	42.4	2.6E+08
1900	25.5	4.6E+10	42.4	5.2E+08
2000	25.6	6.7E+10	42.4	9.7E+08

Table S3. Free-energy barriers to H ejection ($\Delta A^{\ddagger}$) from variational transition-state theory and corresponding H-ejection rate constants (from Eyring equation) at various temperatures for $C_{13}H_{11}$ (**IV**) from the ZZ and FE sites.

	ZZ ejection		FE ejection	
Temperature (K)	$\Delta A^{\ddagger}$ (kcal/mol)	Rate const. (s^{-1})	$\Delta A^{\ddagger}$ (kcal/mol)	Rate const. (s^{-1})
500	30.7	3.8E-01	72.8	1.6E-19
600	30.7	8.2E+01	72.4	5.5E-14
700	30.7	3.9E+03	71.9	5.2E-10
800	30.6	7.1E+04	71.5	5.0E-07
900	30.6	6.9E+05	71.0	1.1E-04
1000	30.6	4.2E+06	70.6	7.8E-03
1100	30.7	1.9E+07	70.2	2.6E-01
1200	30.7	6.5E+07	69.7	5.0E+00
1300	30.7	1.9E+08	69.3	6.0E+01
1400	30.7	4.6E+08	69.0	5.0E+02
1500	30.8	1.0E+09	68.6	3.2E+03
1600	30.8	2.1E+09	68.2	1.6E+04
1700	30.9	3.8E+09	67.9	6.6E+04
1800	30.9	6.6E+09	67.6	2.3E+05
1900	31.0	1.1E+10	67.3	7.2E+05
2000	31.1	1.7E+10	67.0	2.0E+06

Table S4. Free-energy barriers to H ejection ($\Delta A^{\ddagger}$) from variational transition-state theory and corresponding H-ejection rate constants (from Eyring equation) at various temperatures for C_9H_9 (**V**) from the ZZ and FE sites.

	ZZ ejection		FE ejection	
Temperature (K)	$A^{\ddagger}$ (kcal/mol)	Rate const. (s^{-1})	$A^{\ddagger}$ (kcal/mol)	Rate const. (s^{-1})
500	43.0	1.8E-06	48.6	6.1E-09
600	42.9	3.0E-03	48.5	2.7E-05
700	42.8	6.3E-01	48.4	1.2E-02
800	42.7	3.6E+01	48.3	1.1E+00
900	42.6	8.3E+02	48.2	3.7E+01
1000	42.6	1.0E+04	48.1	6.3E+02
1100	42.5	8.3E+04	48.1	6.5E+03
1200	42.5	4.6E+05	48.0	4.6E+04
1300	42.5	2.0E+06	47.9	2.4E+05
1400	42.4	6.9E+06	47.9	9.9E+05
1500	42.4	2.1E+07	47.8	3.4E+06
1600	42.4	5.4E+07	47.8	9.8E+06
1700	42.4	1.3E+08	47.8	2.5E+07
1800	42.4	2.7E+08	47.8	5.8E+07
1900	42.4	5.2E+08	47.9	1.2E+08
2000	42.4	9.6E+08	47.9	2.4E+08

Table S5.
Free-energy barriers to H ejection ($\Delta A^{\ddagger}$) from variational transition-state theory and corresponding H-ejection rate constants (from Eyring equation) at various temperatures for C_9H_9 isomers.

	**I** ZZ ejection		**I** FE ejection		**III** FE ejection	
Temperature (K)	$\Delta A^{\ddagger}$ (kcal/mol)	Rate const. (s^{-1})	$\Delta A^{\ddagger}$ (kcal/mol)	Rate const. (s^{-1})	$\Delta A^{\ddagger}$ (kcal/mol)	Rate const. (s^{-1})
500	22.8	1.2E+03	51.0	5.1E-10	27.9	7.0E+00
600	22.8	6.2E+04	50.9	3.5E-06	27.9	8.4E+02
700	22.8	1.1E+06	50.8	2.0E-03	28.0	2.6E+04
800	22.8	9.6E+06	50.8	2.3E-01	28.1	3.6E+05
900	22.9	5.3E+07	50.6	9.5E+00	28.1	2.8E+06
1000	22.9	2.1E+08	50.5	1.9E+02	28.2	1.4E+07
1100	22.9	6.5E+08	50.4	2.2E+03	28.3	5.4E+07
1200	23.0	1.6E+09	50.3	1.7E+04	28.5	1.6E+08
1300	23.0	3.6E+09	50.3	9.5E+04	28.6	4.2E+08
1400	23.1	7.2E+09	50.3	4.2E+05	28.7	9.6E+08
1500	23.2	1.3E+10	50.2	1.5E+06	28.9	1.9E+09
1600	23.3	2.2E+10	50.2	4.7E+06	29.0	3.6E+09
1700	23.4	3.5E+10	50.2	1.3E+07	29.2	6.3E+09
1800	23.5	5.3E+10	50.1	3.1E+07	29.3	1.0E+10
1900	23.6	7.6E+10	50.1	6.8E+07	29.5	1.6E+10
2000	23.7	1.1E+11	50.1	1.4E+08	29.6	2.4E+10

Table S6. Free-energy barriers to H ejection ($\Delta A^{\ddagger}$) from variational transition-state theory and corresponding H-ejection rate constants (from Eyring equation) for indene, cyclopentadiene, and propyne.

	Indene		Cyclopentadiene		Propyne	
Temperature (K)	$\Delta A^{\ddagger}$ (kcal/mol)	Rate const. (s^{-1})	$\Delta A^{\ddagger}$ (kcal/mol)	Rate const. (s^{-1})	$\Delta A^{\ddagger}$ (kcal/mol)	Rate const. (s^{-1})
500	76.9	2.5E-21	77.7	1.2E-21	86.5	1.6E-25
600	76.4	1.8E-15	77.1	1.1E-15	85.8	6.9E-19
700	75.9	2.9E-11	76.5	1.9E-11	85.1	4.0E-14
800	75.4	4.1E-08	75.9	3.1E-08	84.3	1.5E-10
900	75.0	1.2E-05	75.4	9.5E-06	83.6	9.3E-08
1000	74.5	1.1E-03	74.8	9.3E-04	82.9	1.6E-05
1100	74.0	4.5E-02	74.3	4.0E-02	82.2	1.1E-03
1200	73.6	9.8E-01	73.8	9.1E-01	81.6	3.5E-02
1300	73.2	1.3E+01	73.3	1.3E+01	80.9	6.7E-01
1400	72.8	1.3E+02	72.9	1.2E+02	80.3	8.5E+00
1500	72.5	8.7E+02	72.4	8.7E+02	79.7	7.7E+01
1600	72.1	4.7E+03	72.0	4.8E+03	79.1	5.3E+02
1700	71.8	2.1E+04	71.7	2.2E+04	78.5	2.8E+03
1800	71.5	7.9E+04	71.3	8.2E+04	78.0	1.3E+04
1900	71.2	2.6E+05	71.0	2.7E+05	77.5	4.9E+04
2000	70.9	7.4E+05	70.7	7.9E+05	77.0	1.6E+05

Table S7. Free-energy barriers to H ejection ($\Delta A^{\ddagger}$) from variational transition-state theory and corresponding H-ejection rate constants (from Eyring equation) for the lowest triplet states of cyclopentadiene and toluene.

	Triplet cyclopentadiene		Triplet toluene	
Temperature (K)	$\Delta A^{\ddagger}$ (kcal/mol)	Rate const. (s^{-1})	$\Delta A^{\ddagger}$ (kcal/mol)	Rate const. (s^{-1})
500	27.0	1.7E+01	27.7	8.3E+00
600	27.2	1.5E+03	28.1	7.6E+02
700	27.5	3.9E+04	28.5	1.9E+04
800	27.7	4.5E+05	28.9	2.1E+05
900	27.9	3.1E+06	29.4	1.4E+06
1000	28.2	1.5E+07	29.9	6.1E+06
1100	28.4	5.3E+07	30.4	2.1E+07
1200	28.6	1.5E+08	30.9	6.0E+07
1300	28.9	3.8E+08	31.4	1.4E+08
1400	29.1	8.3E+08	31.9	3.1E+08
1500	29.4	1.6E+09	32.4	6.0E+08
1600	29.7	3.0E+09	32.9	1.1E+09
1700	29.9	5.1E+09	33.4	1.8E+09
1800	30.2	8.2E+09	34.0	2.8E+09
1900	30.4	1.3E+10	34.5	4.3E+09
2000	30.7	1.8E+10	35.1	6.2E+09

Table S8. Free-energy barriers to H ejection ($\Delta A^{‡}$) from variational transition-state theory and corresponding H-ejection rate constants (from Eyring equation) for H ejection from cyclopentadiene, using electronic energies from ωB97M-V/def2-TZVPD and ωB97M(2)/aug-cc-pVTZ.

	ωB97M-V/def2-TZVPD		ωB97M(2)/aug-cc-pVTZ	
Temperature (K)	$\Delta A^{‡}$ (kcal/mol)	Rate const. (s^{-1})	$\Delta A^{‡}$ (kcal/mol)	Rate const. (s^{-1})
500	77.7	1.2E-21	77.1	2.1E-21
600	77.1	1.1E-15	76.6	1.6E-15
700	76.5	1.9E-11	76.0	2.7E-11
800	75.9	3.1E-08	75.5	4.0E-08
900	75.4	9.5E-06	75.0	1.2E-05
1000	74.8	9.3E-04	74.5	1.1E-03
1100	74.3	4.0E-02	74.0	4.5E-02
1200	73.8	9.1E-01	73.6	9.9E-01
1300	73.3	1.3E+01	73.2	1.4E+01
1400	72.9	1.2E+02	72.8	1.3E+02
1500	72.4	8.7E+02	72.4	8.8E+02
1600	72.0	4.8E+03	72.1	4.8E+03
1700	71.7	2.2E+04	71.8	2.1E+04
1800	71.3	8.2E+04	71.5	7.9E+04
1900	71.0	2.7E+05	71.2	2.6E+05
2000	70.7	7.9E+05	71.0	7.4E+05

Table S9. Free-energy barriers to H ejection ($\Delta A^{\ddagger}$) from variational transition-state theory and corresponding H-ejection rate constants (from Eyring equation) for H ejection from the ZZ site of **I**, using electronic energies from ωB97M-V/def2-TZVPD and ωB97M(2)/aug-cc-pVTZ.

	ωB97M-V/def2-TZVPD		ωB97M(2)/aug-cc-pVTZ	
Temperature (K)	$\Delta A^{\ddagger}$ (kcal/mol)	Rate const. (s^{-1})	$\Delta A^{\ddagger}$ (kcal/mol)	Rate const. (s^{-1})
500	22.8	1.2E+03	21.7	3.6E+03
600	22.8	6.2E+04	21.7	1.5E+05
700	22.8	1.1E+06	21.8	2.3E+06
800	22.8	9.6E+06	21.9	1.8E+07
900	22.9	5.3E+07	21.9	8.9E+07
1000	22.9	2.1E+08	22.0	3.2E+08
1100	22.9	6.5E+08	22.1	9.5E+08
1200	23.0	1.6E+09	22.1	2.3E+09
1300	23.0	3.6E+09	22.2	5.0E+09
1400	23.1	7.2E+09	22.3	9.7E+09
1500	23.2	1.3E+10	22.4	1.7E+10
1600	23.3	2.2E+10	22.5	2.8E+10
1700	23.4	3.5E+10	22.6	4.5E+10
1800	23.5	5.3E+10	22.7	6.6E+10
1900	23.6	7.6E+10	22.8	9.5E+10
2000	23.7	1.1E+11	22.9	1.3E+11

Table S10. RRKM microcanonical rate constants (in s^{-1}) for H ejection from selected C_9H_9 isomers, arising from vinylcyclopentadienyl and acetylene at 1600 K (and subsequent isomerization). The 0 K bond-dissociation energy (BDE) is also provided.

Species	Rate constant with average vibrational energy $k(\langle E \rangle)$ ($\langle E \rangle = \int E\, p(E) dE$)		Rate constant integrated over all energies $\langle k \rangle = \int k(E)\, p(E) dE$		BDE (kcal/mol)
	Cutoff 100 cm^{-1}	Cutoff 500 cm^{-1}	Cutoff 100 cm^{-1}	Cutoff 500 cm^{-1}	
**I** ZZ	7.3E+10	1.5E+11	8.6E+10	1.7E+11	13.9
**III** FE	2.2E+10	6.2E+10	2.8E+10	7.7E+10	21.3
**V** ZZ	2.3E+08	3.3E+08	5.6E+08	8.4E+08	38.8
**VII** FE	8.7E+08	2.8E+09	1.4E+09	4.6E+09	40.5
**VIII** FE	3.4E+09	1.1E+10	4.7E+09	1.6E+10	31.4

Table S11. RRKM microcanonical rate constants (in s^{-1}) for H ejection from selected C_9H_9 isomers, arising from benzyl and acetylene at 1600 K (and subsequent isomerization). The 0 K bond dissociation energy (BDE) is also provided.

Species	Rate constant with average vibrational energy $k(\langle E \rangle)$ ($\langle E \rangle = \int E\, p(E) dE$)		Rate constant integrated over all energies $\langle k \rangle = \int k(E)\, p(E) dE$		BDE (kcal/mol)
	Cutoff 100 cm^{-1}	Cutoff 500 cm^{-1}	Cutoff 100 cm^{-1}	Cutoff 500 cm^{-1}	
**I** ZZ	2.6E+10	5.3E+10	3.6E+10	7.2E+10	13.9
**III** FE	7.1E+09	2.0E+10	1.1E+10	3.0E+10	21.3
**V** ZZ	2.2E+07	3.0E+07	1.2E+08	1.9E+08	38.8
**VII** FE	1.9E+08	5.7E+08	4.1E+08	1.3E+09	40.5
**VIII** FE	8.6E+08	2.8E+09	1.6E+09	5.3E+09	31.4

Table S12.
Arrhenius parameters for H-ejection rate constants for indene, cyclopentadiene, and propyne.[a]

Species	A	n	$\frac{E}{R}$ (K)	Source
Indene	9.94E+14	-0.122	40180	This work
Indene	4.95E+14	0.148	40780	da Silva & Bozzelli (1)
Cyclopentadiene	6.07E+14	-0.0171	40660	This work
Cyclopentadiene	1.61E+13	0.86	45080	Harding et al. (4)[b]
Cyclopentadiene	1.0E+19	-0.655	44260	Kern et al. (6)
Cyclopentadiene	1.1E+15	0	38750	Laskin and Lifschitz (7)
Cyclopentadiene (1 atm)	3.236E+49	-10.009	50920	Robinson and Lindstedt (5)
Cyclopentadiene (∞)[c]	3.031E+18	-0.532	43430	Robinson and Lindstedt (5)
Cyclopentadiene	1.730E+68	-15.160	58530	Narayanaswami et al. (8)
Cyclopentadiene	4.0E+14	0	38760	Roy et al. (9)
Cyclopentadiene	1.55E+18	-0.8	42300	Tokmakov et al. (10)
Propyne	9.50E+14	0.0373	45550	This work
Propyne	2.58E+41	-7.81	50590	Giri et al. (13)

[a]The Arrhenius expression is given as $k(\mathrm{s}^{-1}) = AT^n \mathrm{e}^{-\frac{E}{R}/T}$, where *k* is the rate constant, *R* is the universal gas constant, *E* is the activation energy, and *T* is temperature in Kelvin.
[b]Values from Harding et al. (4), as given by Robinson and Lindstedt (5).
[c]The rate constant is given for the high-pressure limit.

Table S13.
Arrhenius parameters for H-abstraction rate constants for indene, cyclopentadiene, and propyne.[a]

Species	*A*	*n*	$\frac{E}{R}$ (K)	Source
Indene	1.1E+05	2.5	-970	Pousse et al. (2)
Cyclopentadiene	1.0E+14	0	2770	Laskin and Lifschitz (7)
Cyclopentadiene	8.587E+13	1.847	1679	Robinson and Lindstedt (5)
Cyclopentadiene	3.03E+08	1.71	1416	Moskaleva and Lin (11)[b]
Cyclopentadiene	2.80E+13	0	1137	Narayanaswami et al. (8)
Cyclopentadiene	5.1E+07	1.9	2013	Vervust et al. (12)
Propyne	6.40 E+07	1.94	3544	Wang et al. (14)
Propyne	3.57E+04	2.825	2779	Miller et al. (15)
Propyne	1.2E+14	0	4940	Rosado-Reyes et al. (16)

[a]The Arrhenius expression is given as $k(\mathrm{cm^3 mol^{-1} s^{-1}}) = AT^n \mathrm{e}^{-\frac{E}{R}/T}$, where *k* is the bimolecular rate constant, *R* is the universal gas constant, *E* is the activation energy, and *T* is temperature in Kelvin.
[b]Values from Moskaleva and Lin (11), as given by Robinson and Lindstedt (5).

Table S14.
Comparison of energies of species relative to reactants and barrier heights from Fig. 4 with those from Mao et al. (17) for the reaction of vinylcyclopentadienyl with acetylene.

Species	Present study (kcal/mol)	Species name from Mao et al. (17)	Mao et al. (17) (kcal/mol)
Barrier to first adduct	21.3	Barrier to W9_2	16.5
First adduct	0.0	W9_2	-4.0
Barrier, adduct to **V**	5.8	Barrier, W9_2 to W9_12	6.7
**V**	-47.9	W9_12	-48.7
Barrier, **V** to **III**	25.9	Barrier, W9_12 to W9_13	24.7
**III**	-56.8	W9_13	-57.8
Barrier, **III** to Indene+H	27.1	Barrier, W9_13 to Indene+H	26.2
Indene + H	-35.5	Indene + H	-37.7

Dataset S1 (separate file). Spreadsheet containing data used to generate figures.

Zenodo repository: https://doi.org/10.5281/zenodo.13307485
Raw Q-Chem output files and scripts for rate computation (Variational TST at constant temperature and RRKM for constant energy)

SI References

1. G. da Silva, J. W. Bozzelli, Indene formation from alkylated aromatics: Kinetics and products of the fulvenallene + acetylene reaction. J. Phys. Chem. A 113, 8971-8978 (2009).
2. E. Pousse, Z. Y. Tian, P. A. Glaude, R. Fournet, F. Battin-Leclerc, A lean methane premixed laminar flame doped with components of diesel fuel part III: Indane and comparison between n-butylbenzene, n-propylcyclohexane and indane. Combust. Flame 157, 1236-1260 (2010).
3. A. Jain, Y. Wang, W. D. Kulatilaka, Simultaneous imaging of H and OH in flames using a single broadband femtosecond laser source. Proc. Combust. Inst. 38, 1813-1821 (2021).
4. L. B. Harding, S. J. Klippenstein, Y. Georgievski, On the combustion reactions of hydrogen atoms with resonance-stabilized hydrocarbon radicals. J. Phys. Chem. A 111, 3789-3801 (2007).
5. K. R. Robinson, R. P. Lindstedt, On the chemical kinetics of cyclopentadiene oxidation. Combust. Flame 158, 666-686 (2011).
6. R. D. Kern et al., Pyrolysis of cyclopentadiene: Rates for initial C-H bond fission and the decomposition of c-C_5H_5. Proc. Combust. Inst. 27, 143-150 (1998).
7. A. Laskin, A. Lifschitz, Thermal decomposition of indene. Experimental results and kinetic modeling. Symp. (Int.) Combust. 27, 313-320 (1998).
8. K. Narayanaswamy, H. Pitsch, P. Pepiot, A chemical mechanism for low to high temperature oxidation of methylcyclohexane as a component of transportation fuel surrogates. Combust. Flame 162, 1193-1213 (2015).
9. K. Roy, C. Horn, P. Frank, V. G. Slutsky, T. Just, High-temperature investigations of the pyrolysis of cyclopentadiene. Proc. Combust. Inst. 27, 329-336 (1998).
10. I. V. Tokmakov, L. V. Moskaleva, M. C. Lin, Quantum chemical/vRRKM study on the thermal decomposition of cyclopentadiene. Int. J. Chem. Kinet. 36, 139-151 (2004).
11. L. V. Moskaleva, M. C. Lin, Unimolecular isomerization/decomposition of cyclopentadienyl and related bimolecular reverse process: Ab initio MO/statistical theroy study. J. Comput. Chem. 21, 415-425 (2000).
12. A. J. Vervust et al., Detailed experimental and kinetic modeling study of cyclopentadiene pyrolysis in the presence of ethene. Energy Fuels 32, 3920-3934 (2018).
13. B. R. Giri, R. X. Fernandes, T. Bentz, H. Hippler, M. O'lzmann, High-temperature kinetics of propyne and allene: Decomposition vs. isomerization. Proc. Combust. Inst. 33, 267-272 (2011).

14. Q.-D. Wang, Y. Sun, M.-M. Sun, J.-H. Liang, Chemical kinetics of hydrogen atom abstraction from propargyl sites by hydrogen and hydroxy radicals. Int. J. Molec. Sci. 20, 3227 (2019).
15. J. A. Miller, J. P. Senosiain, S. J. Klippenstein, Y. Georgievski, Reactions over multiple, interconnected potential wells: Unimolecular and bimolecular reactions on a C_3H_5 potential. J. Phys. Chem. A 112, 9429-9438 (2008).
16. C. M. Rosado-Reyes, J. A. Manion, W. Tsang, Kinetics of the thermal reaction of H atoms with propyne. J. Phys. Chem. A 114, 5710-5717 (2010).
17. Q. Mao, L. Cai, R. Langer, H. Pitsch, The role of resonance-stabilized radical chain reactions in polycyclic aromatic hydrocarbon growth: Theoretical calculation and kinetic modeling. Proc. Combust. Inst. 38, 1459-1466 (2021).
